# Cancer-associated fibroblasts induce PDL1+ neutrophils through the IL6-STAT3 pathway that foster immune suppression in hepatocellular carcinoma

**DOI:** 10.1038/s41419-018-0458-4

**Published:** 2018-03-19

**Authors:** Yusheng Cheng, Hui Li, Yinan Deng, Yan Tai, Kaining Zeng, Yingcai Zhang, Wei Liu, Qi Zhang, Yang Yang

**Affiliations:** 1 Department of Hepatic Surgery, The Third Affiliated Hospital of Sun Yat-sen University, Guangzhou, China; 2 Guangdong Provincial Key Laboratory of Liver Disease Research, Guangzhou, China; 3 Cell-Gene Therapy Translational Medicine Research Center, The Third Affiliated Hospital of Sun Yat-sen University, Guangzhou, China

## Abstract

Emerging evidence indicate that cancer-associated fibroblasts (CAFs) affect tumor progression by reshaping the tumor microenvironment. Neutrophils are prominent components of solid tumors and important in cancer progression. Whether the phenotype and function of neutrophils in hepatocellular carcinoma (HCC) are influenced by CAFs is not well understood. Herein, we investigated the effect of HCC-derived CAFs (HCC-CAFs) on the neutrophils and explored the biological role of this effect. We found that HCC-CAFs induced chemotaxis of neutrophils and protected them from spontaneous apoptosis. Neutrophils were activated by the conditioned medium from HCC-CAFs with increased expression of CD66b, PDL1, IL8, TNFa, and CCL2, and with decreased expression of CD62L. HCC-CAF-primed neutrophils impaired T-cell function through the PD1/PDL1 signaling pathway. We revealed that HCC-CAFs induced the activation of STAT3 pathways in neutrophils, which are essential for the survival and function of activated neutrophils. In addition, we demonstrated that HCC-CAF-derived IL6 was responsible for the STAT3 activation of neutrophils. Collectively, our results suggest that HCC-CAFs regulate the survival, activation, and function of neutrophils within HCC through an IL6–STAT3–PDL1 signaling cascade, which presents a novel mechanism for the role of CAFs in remodeling the cancer niche and provides a potential target for HCC therapy.

## Introduction

Neutrophils, as the most abundant leucocytes, are also one of the most infiltrated immune cells within the tumors. Emerging evidence has shown that neutrophils are critical for tumor initiation and progression^1,2^. The increased presence of tumor infiltrated neutrophils has been linked to a poorer prognosis for patients with solid tumors^2^. Neutrophils promote genetic mutations leading to tumorigenesis or promote tumor cell proliferation^3^, tumor vascularization, migration, and invasion^4–6^ via several factors. In addition, neutrophils also mediate the tumor immune evasion by suppressing antitumor immunity^7^.

The infiltration of neutrophils into tumors has been shown to be mediated by factors produced by tumor cells. Tumor-derived CXCL5 modulates the recruitment and activation of neutrophils, which in turn enhances the migration and invasion of human hepatocellular carcinoma (HCC) cells^8^. Head and neck squamous cell carcinoma (HNSCC) cell-derived MIF induces the chemotaxis and activation of neutrophils through a p38-dependent mechanism^9^. Neutrophils respond to hyaluronan fragments from HCC cell supernatants via PI3K/Akt signaling, which leads to prolonged survival and a stimulating effect on HCC cell motility^10^. IL-17 promotes the migration of neutrophils into HCC through epithelial cell-derived CXC chemokines, leading to increased MMP-9 production and angiogenesis at the tumor edge^11^. However, much less is known about the role of stromal cells in modulating the phenotype and function of neutrophils within tumors thus far.

Cancer-associated fibroblasts (CAFs), which are a major component of the tumor stroma, are often exposed to distinct inflammatory cells and factors within the tumor microenvironment^12^, therefore, they may acquire novel functions^13–18^ that are not present in normal fibroblasts, and these unique functions may have a role in remodeling the tumor microenvironment and ultimately affect tumor progression. As neutrophils are one type of the most infiltrated immune cells within the HCC, it is possible that an intense interaction may exist between the HCC-derived CAFs (HCC-CAFs) and the infiltrated neutrophils.

## Results

### HCC-CAFs recruit peripheral blood neutrophils by the SDF1a/CXCR4 pathway

**Fig. 1:**
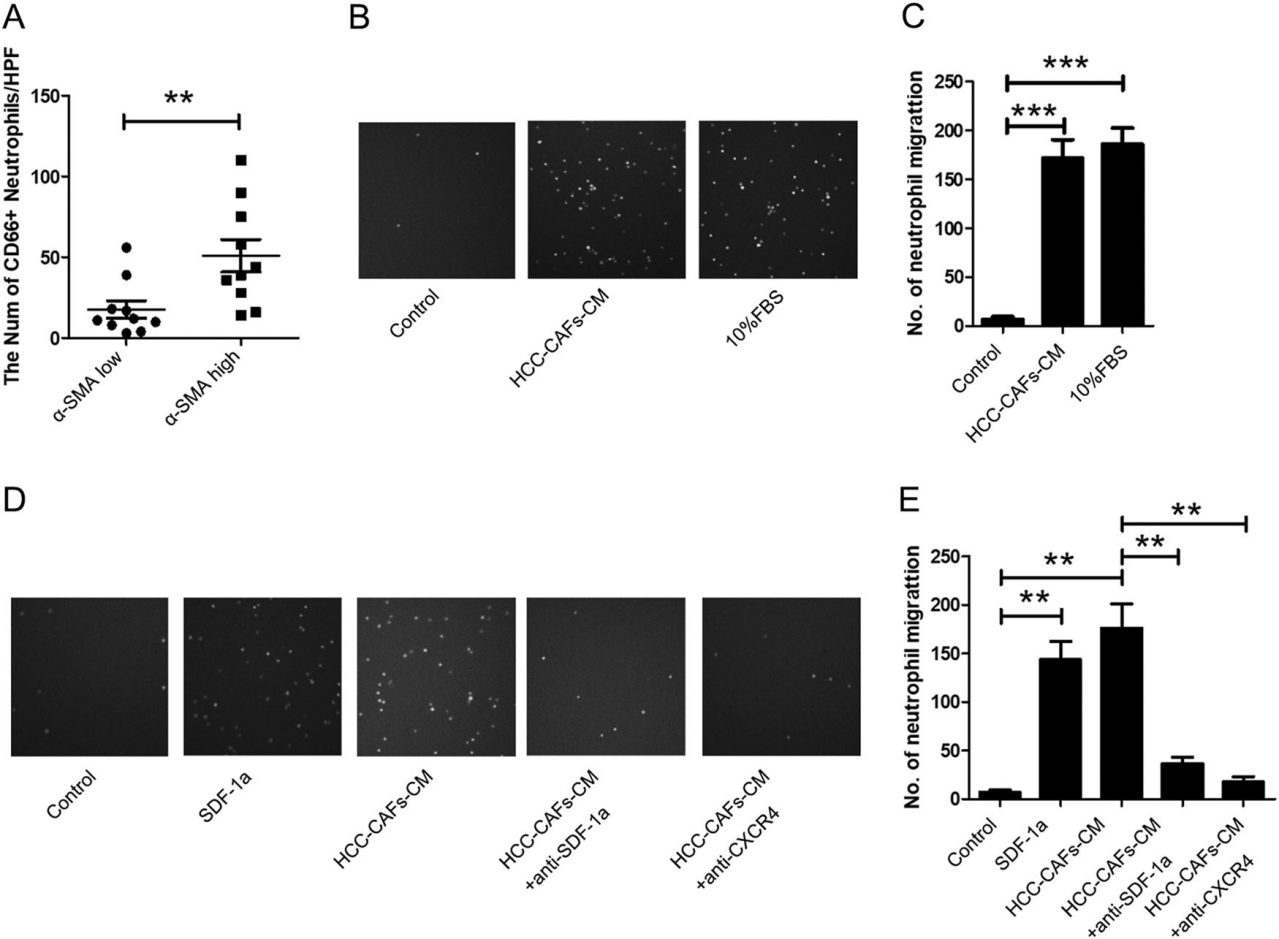
HCC-CAFs attract neutrophils via the SDF1a/CXCR4 pathway. **a** HCC samples were stained with CD66b and α-SMA antibodies. Hepatoma samples were divided into two groups according to the density of α-SMA density: high density (*n* = 10) and low density (*n* = 10). Correlation between levels of α-SMA and CD66b were analyzed, **P < 0.01. **b**, **c** Neutrophils isolated from normal donor peripheral blood samples were serum-starved and then allowed to migrate for 3 h toward CM from HCC-CAFs. **b** One representative experiment is shown. **c** The data indicate the mean ± SEM of three independent experiments, **P < 0.01. **d**, **e** Neutrophils isolated from normal donor peripheral blood samples were serum-starved and then allowed to migrate for 3 h toward HCC-CAFs CM in the presence or absence of CXCR4 or SDF1a blocking antibodies. **d** One representative experiment is shown. **e** The data indicate the mean ± SEM of three independent experiments, **P < 0.01

We examined the distribution of CD66b+ neutrophils and CAFs in HCC samples and confirmed that there is a positive correlation between the densities of CAFs and CD66b+ neutrophils (Fig. 1a). To determine the effect of HCC-CAFs on neutrophil recruitment, cell chemotaxis assays were performed in 24-well Transwell chambers. As shown in Fig. 1b, c, neutrophils were more attracted to HCC-CAFs conditioned medium (HCC-CAFs CM) than to control medium. Our previous studies showed that HCC-CAFs recruit peripheral blood monocytes by SDF1a^19^, therefore, we explored the roles of this factor in neutrophil chemotaxis. We observed that CXCR4 blocking antibody was able to reduce the migration of neutrophils induced by HCC-CAFs CM (Fig. 1d, e). Moreover, we observed that SDF1a could induce the migration of neutrophils and that treatment with SDF1a blocking antibodies was able to inhibit the migration of neutrophils (Fig. 1d, e). Therefore, HCC-CAFs attract peripheral blood neutrophils through the SDF1a/CXCR4 pathway.

### HCC-CAFs sustain neutrophil survival and activation through the JAK-STAT3 signaling pathway

**Fig. 2:**
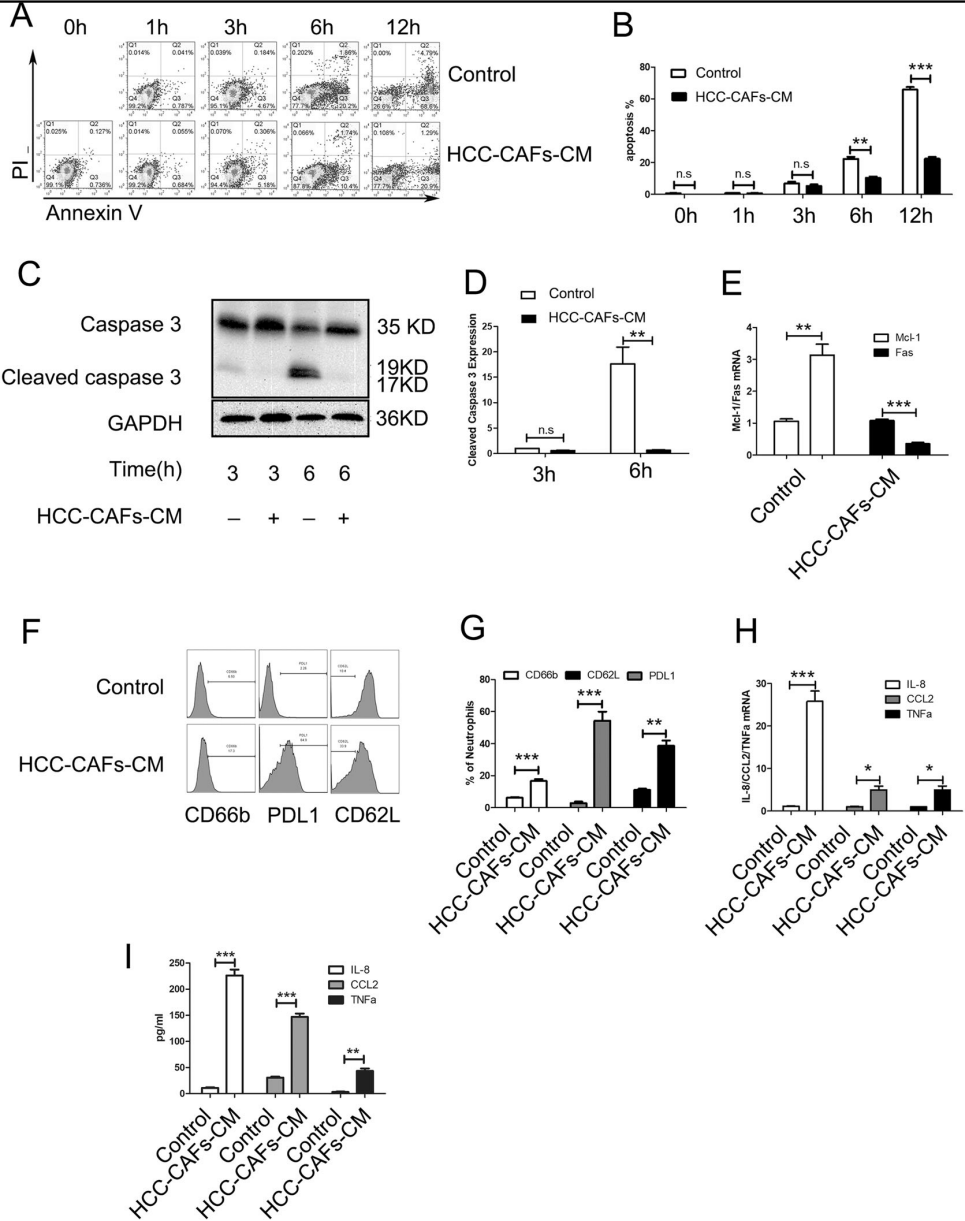
**HCC-CAFs sustain neutrophil survival and activation.a**, **b** Neutrophils were incubated in the presence or absence of HCC-CAFs CM for 0, 1, 3, 6, or 12 h, the apoptosis of neutrophils was determined by using an Annexin V/PI apoptosis detection kit. Representative FACS plots of the apoptotic rate are shown in **a**. Frequencies of apoptotic neutrophils are shown in **b**. **c**, **d** Neutrophils were incubated in the presence or absence of HCC-CAFs CM for 3 or 6 h. Total protein was extracted from neutrophils and the levels of Caspase 3 were detected by using western blotting. GAPDH was used as a loading control. **c** One representative experiment is shown. **d** The data indicate the mean ± SEM of three independent experiments, **P < 0.01. **e** Neutrophils were incubated in the presence or absence of HCC-CAFs CM for 6 h, and total RNA was extracted from neutrophils and real-time PCR was performed to determine the mRNA levels of Mcl-1 and FAS, *P < 0.05. **f**, **g** Neutrophils were incubated in the presence or absence of HCC-CAFs CM for 6 h. The expression of CD66b, PDL1, and CD62L on neutrophils was determined by flow cytometry. **f** One representative experiment is shown. **g** The data indicate the mean ± SEM of three independent experiments, **P < 0.01, *P < 0.05. **h** Neutrophils were incubated in the presence or absence of HCC-CAFs CM for 6 h. Total RNA was extracted from neutrophils and real-time PCR was performed to determine them RNA levels of IL8, CCL2, and TNFa, ***P < 0.001, *P < 0.05. **i** Neutrophils were incubated in the presence or absence of HCC-CAFs CM for 48 h, and CM was collected and ELISA was performed to determine the protein levels of IL8, CCL2, and TNFa, ***P < 0.001, **P < 0.01

To identify the effect of HCC-CAFs on neutrophil survival, human peripheral blood neutrophils were cultured in the presence or absence of the conditioned medium from HCC-CAFs for different lengths of time and were harvested and stained with Annexin V/PI to evaluate the apoptosis. The results of flow cytometry showed that HCC-CAFs CM significantly reduced the percentage of apoptotic neutrophils (Fig. 2a, b). To further confirm this result, neutrophils treated with HCC-CAFs CM were collected and cell lysates were prepared for immunoblotting assay. The results of the western blot showed that the level of cleaved caspase 3 decreased in neutrophils treated with HCC-CAFs CM (Fig. 2c, d). The results of quantitative PCR showed that HCC-CAFs CM treatment up-regulated the expression of Mcl-1 but downregulated that of Fas in neutrophils (Fig. 2e), which suggests that HCC-CAFs CM has a protective effect on neutrophils.

To demonstrate whether HCC-CAFs could also promote the activation of neutrophils, we investigated the expression of IL8, CCL2, TNFa, CD66b, PDL1, and CD62L in neutrophils incubated with or without HCC-CAFs CM. Treatment with HCC-CAFs CM significantly upregulated the mRNA and protein levels of IL-8, CCL2, and TNFa in neutrophils (Fig. 2h, i). Flow cytometry showed that HCC-CAFs CM significantly increased the expression of CD66b and PDL1, but decreased that of CD62L in neutrophils (Fig. 2f, g), suggesting that neutrophils were activated by HCC-CAFs.

**Fig. 3:**
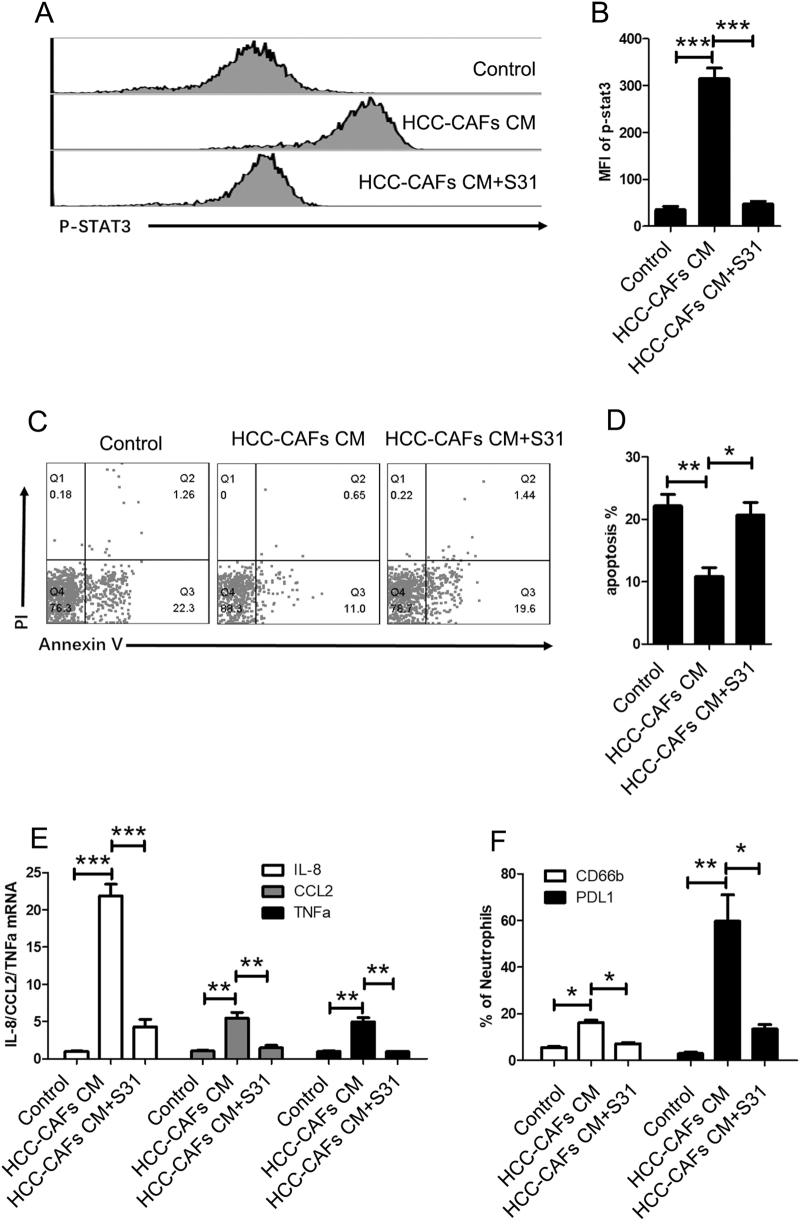
**HCC-CAFs sustain neutrophil survival and activation via the JAK-STAT3 pathway.a**, **b** Neutrophils were cultured on HCC-CAFs CM in the presence or absence of S31, and the phosphorylation level of STAT3 in neutrophils was analyzed by flow cytometry. **a** One representative experiment is shown. **b** The data indicate the mean ± SEM of three independent experiments, *P < 0.05. **c**, **d** Neutrophils were cultured on HCC-CAFs CM in the presence or absence of S31. The apoptosis of neutrophils was determined by using an Annexin V/PI apoptosis detection kit. Representative FACS plots of the apoptotic rate are shown in **c**. Frequencies of apoptotic neutrophils are shown in **d. e** Total RNA was extracted from neutrophils and real-time PCR was performed to determine them RNA levels of IL8, CCL2, and TNFa, ***P < 0.001, **P < 0.01, *P < 0.05. **f** The CD66b, PDL1, and CD62L expression on neutrophils was determined by flow cytometry, **P < 0.01, *P < 0.05

To determine the signaling pathways that are responsible for the survival and activation of neutrophils by HCC-CAFs CM, human peripheral blood neutrophils were incubated with HCC-CAFs CM for 30 min. Flow cytometry analyses demonstrated that STAT3 was strongly activated after stimulation (Fig. 3a, b). We next tested whether the survival and activation of neutrophils was modulated by HCC-CAFs CM through the JAK-STAT3 signal pathway. For this aim, we stimulated neutrophils with HCC-CAFs CM in the presence or absence of the STAT3 inhibitor S31 (10 µg/mL) and then evaluated the rate of apoptosis in neutrophils. The flow cytometry showed that S31 increased the rate of apoptosis in neutrophils (Fig. 3c, d), suggesting that HCC-CAFs CM protects neutrophils from apoptosis through the JAK-STAT3 signaling pathway. Quantitative PCR and flow cytometry analyses showed that treatment with HCC-CAFs CM in the presence of S31 reversed the enhancement of IL-8, TNFa, and CCL2 mRNA levels and CD66b and PDL1 protein levels in neutrophils (Fig. 3e, f), suggesting that the activation of neutrophils by HCC-CAFs CM is dependent on the JAK-STAT3 signaling pathway. In summary, these results indicate that the JAK-STAT3 signal pathway is responsible for HCC-CAF-primed neutrophil survival and activation.

### HCC-CAF-primed neutrophils suppress T-cell immunity through the STAT3-PDL1 signaling pathway

**Fig. 4:**
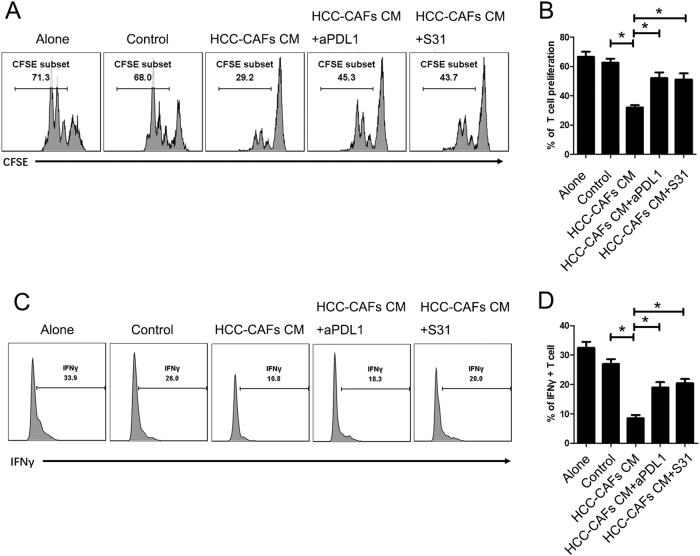
**HCC-CAF-primed neutrophils suppress T-cell immunity through the STAT3-PDL1 signaling pathway.** Neutrophils were cultured on HCC-CAFs CM as described previously. Isolated 5,6-carboxyfluorescein (CSFE)-labeled CD3+ T cells were stimulated using anti-CD3/CD28 mAb beads, and neutrophils were added at a 1:1 ratio in the presence or absence of aPDL1 or S31. After 3 days, these cells were collected and stained and labeled with anti-CD3 antibody. The proliferation (**a**, **b**) and IFNγ expression (**c**, **d**) of CD3+ T cell were analyzed by flow cytometry. **a**, **c** One representative experiment is shown. **b**, **d** The data indicate the mean ± SEM of three independent experiments, *P < 0.05

To identify the effect of HCC-CAF-primed neutrophils on T-cell function, purified peripheral CD3+ T cells were co-cultured with HCC-CAF-primed autologous blood neutrophils. Interestingly, HCC-CAF-primed neutrophils efficiently suppressed IFNγ production and proliferation of T cells (Fig. 4a–d). To determine whether PDL1 operates on the suppression of T-cell function, we added PDL1 neutralizing antibodies to T cell/HCC-CAF-primed neutrophil co-culture. Interestingly, blockage of PDL1 significantly attenuated such T cell suppression mediated by HCC-CAF-primed neutrophils (Fig. 4a–d). These results above indicate that, in the HCC environment, neutrophils are activated by fibroblast-derived soluble factors and acquire the ability to suppress T-cell function through the PD1/PDL1 pathway.

To test whether STAT3 signaling is also involved in the immune suppression function of neutrophil, we stimulated neutrophils with HCC-CAFs CM in the presence or absence of 10 µg/mL S31. As shown in Fig. 4a–d, pre-incubation with S31 greatly impaired the strong immune function of HCC-CAFs CM-primed neutrophils, indicating that STAT3 signaling is an important regulator of neutrophil functions upon stimulation with HCC-CAFs CM.

As shown in Fig. 3f, HCC-CAFs CM significantly increased the PDL1 expression and the STAT3 inhibitor S31 reversed the HCC-CAF-mediated PDL1 expression, which suggests that HCC-CAFs upregulate PDL1 expression by activating STAT3. Therefore, these results indicate that HCC-CAF-primed neutrophils suppress T-cell immunity through the STAT3-PDL1 signaling pathway.

### IL6-STAT3 signaling is crucial for HCC-CAF-mediated activation of neutrophils

**Fig. 5:**
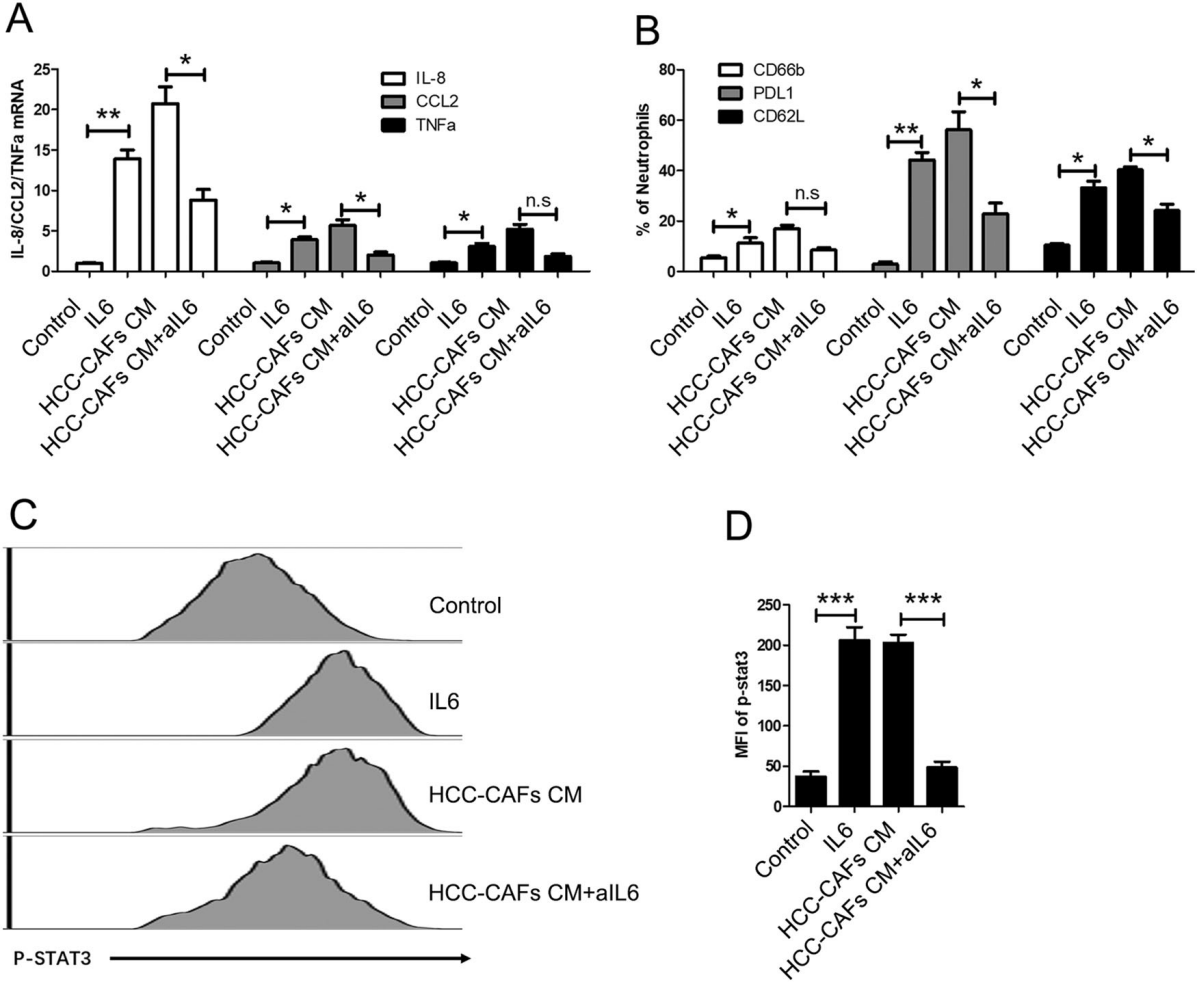
**IL6-STAT3 signaling is crucial for the activation of neutrophil.a** Neutrophils were cultured with IL6 or HCC-CAFs CM in the presence or absence of aIL6. Total RNA was extracted from neutrophils and real-time PCR was performed to determine them RNA levels of IL8, CCL2, and TNFa, **P < 0.01, *P < 0.05. **b** Neutrophils were cultured with IL6 or HCC-CAFs CM in the presence or absence of aIL6. The CD66b, PDL1, and CD62L expression in neutrophils was determined by flow cytometry, **P < 0.01, *P < 0.05. **c**, **d** Neutrophils were cultured with IL6 or HCC-CAFs CM in the presence or absence of aIL6, and the phosphorylation level of STAT3 in neutrophils was analyzed by flow cytometry. **c** One representative experiment is shown. **d** The data indicate the mean ± SEM of three independent experiments, *P < 0.05

Our previous studies showed that IL6 was found to be strongly expressed in HCC-CAFs and was responsible for the HCC-CAF-mediated production of MDSCs. Therefore, we explored the roles of this factor in the activation of neutrophils. To investigate whether the in vitro effects of HCC-CAFs CM on neutrophils were mediated by IL6, we stimulated neutrophils with HCC-CAFs CM in the presence of aIL6 blocking antibody (aIL6). The results of real-time PCR and flow cytometry analyses showed that the blockade of IL6 in neutrophils significantly inhibited the upregulation of IL8, CCL2, and TNFa mRNA and CD66b and PDL1 protein levels induced by HCC-CAFs CM (Fig. 5a, b). Moreover, IL6 alone could significantly increase the expression of IL8, CCL2, TNFa, and PDL1 in neutrophils (Fig. 5a, b).

Given that STAT3 was essential for neutrophil survival, activation and function, we investigated whether IL6 could induce to the STAT3 activation. We collected and analyzed the phosphorylation level of STAT3 in neutrophils by flow cytometry. As shown in Fig. 5c, d, both HCC-CAFs CM and IL6 significantly increased the phosphorylation of STAT3, and blocking IL6 significantly reduced HCC-CAFs CM-mediated STAT3 phosphorylation. These data strongly indicate that IL6-STAT3 signaling is crucial for HCC-CAF-mediated activation of neutrophils.

## Discussion

Here we found that HCC-CAFs induced the chemotaxis of neutrophils and protected them from spontaneous apoptosis. Neutrophils were activated by the conditioned medium from HCC-CAFs, which lead to increased expression of CD66b, PDL1, IL8, TNFa, and CCL2, and with decreased expression of CD62L. HCC-CAF-primed neutrophils impaired T cell immunity through the PD1/PDL1 signaling pathway. We revealed that HCC-CAFs stimulated the activation of the STAT3 pathways in neutrophils, which was essential for the functions of activated neutrophils. In addition, we demonstrated that HCC-CAF-derived IL6 was responsible for the STAT3 activation of neutrophils. Although neutrophil activation has already been described in patients with solid tumors^20^, our study is the first to demonstrate that HCC-CAF-derived IL6 induces PDL1+ neutrophils via the JAK-STAT3 pathway, which impairs T-cell function through PD1/PDL1 signaling, and then creates favorable conditions for HCC progression.

Tumors have long been regarded as wounds that do not heal^21^. During this process, the fibroblasts are “educated” by the tumor to acquire tumor-promoting activities. Accumulating evidence has shown that the CAFs influence the growth, progression, and outcome of cancer cells^15–18^. Our study and recent studies have shown that CAFs have an unexpected regulatory effect on the function of immune cell within the tumor microenvironment^14,18,19,22^. Based on our findings, we propose that HCC-CAFs induce immunosuppressive neutrophils to impair T-cell function mechanistically connecting the pathological role of HCC-CAFs within the tumor microenvironment.

Immunosuppression has been known to be a hallmark of cancer^23^. Crosstalk between PDL1 and PD1 is one of the best-studied and most clinically successful immune-checkpoint drug targets. Neutrophils’ PDL1 upregulation in neutrophils occurs in the context of inflammation or infection^24^. Although previous studies have shown that tumor-activated neutrophils could inhibit T-cell proliferation via Mac-1^25^, we now report that the induced high PDL1 expression levels in neutrophils exhibit profound suppressive role on T-cell proliferation and IFNγ production, which is consistent with the Wang’s observation in gastral cancer^7^. Our results further emphasize the importance of the PD1/PDL1 pathway in tumor-related immunosuppression.

Neutrophils exhibit distinct phenotypes in different tumor microenvironments, and researchers have reported the existence of N1 and N2 neutrophils^26^. N2 neutrophils promote tumour progression mainly through matrix metalloproteinases^27^ and angiogenic factors^28^, which can be prevented by blocking those factors and then lead to a display of the N1 phenotype^26^. However, the tumor-alleviating or tumor-promoting roles of neutrophils have been controversial. In stages of lung cancer, an activating effect of neutrophils on T cells was reported^29^. In contrast, in gastric cancer, neutrophils promote disease progression by inhibiting T-cell function^7^. Our results are consistent with the latter study by showing tumor-promoting effect of neutrophils in HCC. Conversely, it could be speculated that at different tumor progression stages infiltrated neutrophils may have different phenotypes and functions.

Neutrophils constitute the common component of infiltrated leukocytes in tumors, and are derived almost entirely from circulating blood neutrophils^30^. Epithelial cell-derived CXC chemokines are the critical mediators that recruit neutrophils into the peritumoral stroma of HCC tissues, where neutrophils promote angiogenesis progression by the secretion of matrix metalloproteinase-9, which accelerates HCC progression^11^. Hyaluronan derived from hepatoma cells reprograms neutrophils into an activated phenotype, which thereby stimulates the metastasis of malignant cells^10^. GM-CSF derived from hepatoma cells could induce neutrophils to secrete HGF, which in turn leads to the migration and invasion of hepatoma cells^31^. Our results show that IL6 derived from HCC-CAFs reprograms neutrophils into an activated phenotype. This change impairs T-cell immunity through the PD1/PDL1 signaling pathway, which reveals a new role for neutrophils in promoting HCC progression.

The molecular mechanisms mediating neutrophil activation and function within the tumor microenvironment are very complex. Wu et al. suggested that tumor-derived hyaluronan fragments activate the PI3K/Akt pathway in neutrophils to prolong their survival and production of inflammatory factors^10^. HCC-derived GM-CSF activate the Erk1/2, p38, and NF-κB pathways in neutrophils to prolong the metastasis of cancer cells^31^. Soluble factors derived from cancer cells, induce a considerable increase in functional LC3 and autophagosomes in neutrophils, which sustains the survival and pro-tumorigenic effects of neutrophils in human HCC^20^. HNSCC supernatants strongly activate p38 and its downstream kinases CREB and p27 in neutrophils to induce the release of pro-tumor factors^9^. These findings identify the molecular mechanisms through which tumor cells modulate the activation of neutrophils. However, this is little demonstration of a statistically significant role of tumor stroma in neutrophil accumulation and activation. Our studies suggest that HCC-CAFs, one of the most important types of tumor stromal cells, can dramatically attract peripheral blood neutrophils via the SDF1a/CXCR4 signaling pathway, and induce their differentiation into PDL1+ neutrophils with a capacity to inhibit T-cell immunity through the IL6-STAT3-PDL1 signaling cascade, which presents a novel mechanism mediating neutrophil activation and function.

**Fig. 6:**
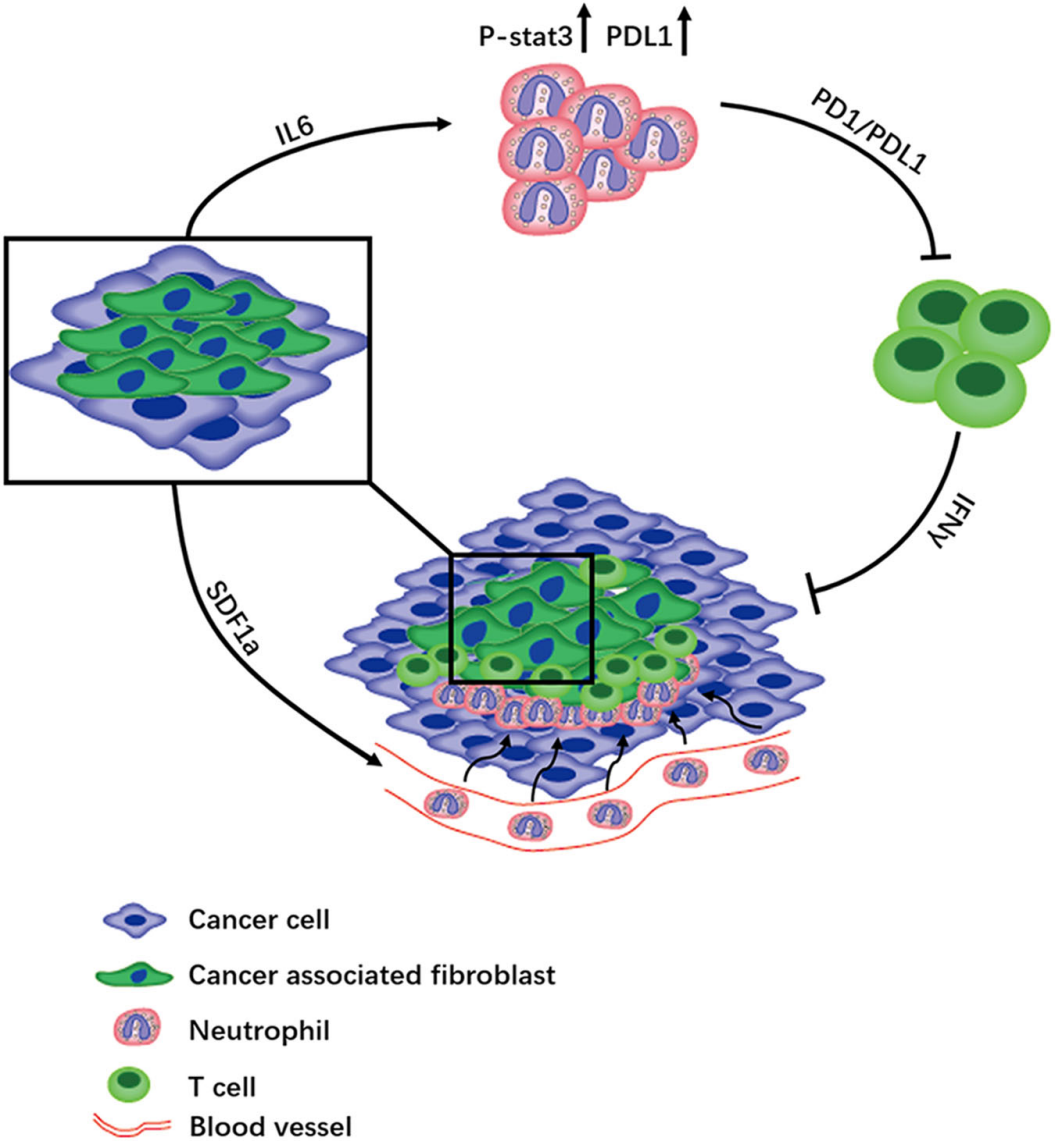
Proposed mechanism of CAF-mediated PDL1+ neutrophils production which foster immune suppression in HCC. Based on our data, we propose a model involving the progressive immunosuppression within HCC. First, HCC-CAFs produce SDF1a which recruits neutrophils into the HCC. Second, released IL6 induces the activation of neutrophils within the tumor microenvironment. This process is accompanied by the induction of PDL1 expression on these cells through the JAK-STAT3 signaling pathway. Third, these activated PDL1+ neutrophils exert a pro-tumor effect by suppressing T cell immunity in a PD1/PDL1-dependent manner within the tumor microenvironment

Based on our data, we propose a model involving the progressive immunosuppression within HCC (Fig. 6). First, HCC-CAFs produce SDF1a which recruits neutrophils into the HCC. Second, IL6 is released, inducing the activation of neutrophils within the tumor microenvironment. This process is accompanied by the induction of PDL1 expression on these cells through the IL6-STAT3 signaling pathway. Third, these activated PDL1+ neutrophils exert a pro-tumor effect by suppressing T-cell immunity in a PD1/PDL1-dependent manner within the tumor microenvironment. In the future, therapeutics aimed at interfering with these pathological neutrophils and the IL6-STAT3-PDL1 immunosuppressive pathway may be developed to provide novel strategies for HCC treatment.

## Materials and methods

### Patient samples and fibroblast isolation and CM collection

Samples from hepatitis B-related HCC were obtained from the third affiliated hospital of Sun Yat-sen University. None of the patients had received anticancer therapy before surgical resection, and patients with concurrent HIV, other cancer or autoimmune diseases were excluded. Human CAFs from hepatitis B-related HCC patients were isolated and fibroblast conditioned medium was collected as described previously^14^.

### Neutrophils isolation

Peripheral blood was collected from healthy volunteers after obtaining written informed consent. Carefully layer 5.0 mL of anti-coagulated whole blood was carefully layered 5.0 mL of PolymorphPrep (Axis-Shield PoC AS, Oslo, Norway) in a 50 mL centrifuge tube. The samples were layered over PolymorPhprep and centrifuged at 800×*g* for 30 min in a swinging bucket rotor at room temperature. After centrifugation, two leukocyte bands were visible. The top band at the sample/medium interface will consisted of mononuclear cells and the lower band consisted of polymorphonuclear cells (PMN). The cell bands were harvested using a Pasteur pipette. The remnant RBC was lysed using a hypotonic lysing procedure to obtain the pure PMN population. Morphological examination and cell count were performed to determine the number and purity of PMN. Neutrophils were cultured in RPMI 1640 (Invitrogen, Carlsbad, CA, USA) supplemented with 10% FBS (fetal bovine serum; Invitrogen) and 1% penicillin/streptomycin. The purity of neutrophils was 98% after this procedure.

### Migration assay

Cell migration assays were performed in 24-well Transwell chambers (Corning Costar, Cambridge, MA) using polycarbonate membranes with a pore size of 5 μm. Approximately 200,000 neutrophils were labeled in 1 mL of PBS containing 10 µM carboxyfluorescein succinimidyl ester (CFSE, Invitrogen, Carlsbad, CA, USA) for 5 min at room temperature and were loaded into the upper chambers. Medium or medium supplemented with 10% FBS, HCC-CAFs CM, SDF1a (50 ng/mL, R&D Systems, Minneapolis, MN), SDF1a blocking antibody(10 μg/mL, R&D Systems, Minneapolis, MN), and CXCR4 blocking antibody was added to the lower chambers. After the cells were incubated for 6 h at 37 °C with 5% CO_2_, the number of cells in the lower chamber was determined.

### Neutrophils activation in fibroblast CM

To study the effect of fibroblast-conditioned medium on neutrophils, the neutrophils were treated with conditioned medium for 48 h. To investigate the activation of neutrophils by fibroblasts, neutrophil cultures were treated with non-toxic levels of STAT3 Inhibitor VI, S3I-201 (S31, 10 µg/mL, Santa, Cruz), or neutralizing IL6 antibodies (mAbs) (10 μg/mL, R&D Systems, Minneapolis, MN).

### Cell apoptosis assay

Neutrophil apoptosis was quantified using an PI/Annexin V apoptosis detection kit according to the manufacturer’s instructions (Invitrogen). The binding of Annexin V-FITC and PI to the cells was measured by FACS Calibur (BD Biosciences, NJ, USA) using Cell Quest software.

### In vitro neutrophil-T-cell co-culture system

To evaluate the suppressive functions of HCC-CAF-primed neutrophils, 5 million CD3+ T cells were labeled in 1 mL of PBS containing 2.5 µM CFSE (Invitrogen, Carlsbad, CA, USA) for 5 min at room temperature and seeded at 100,000 cells per well in a 96-well plate in 100 µL of RPMI 1640 medium containing 10% FBS. HCC-CAFs-primed neutrophils were added at a ratio of 1:1 (100,000 cells) in the presence or absence of a neutralizing antibody against human PDL1 (20 μg/mL). Next, the T cells were activated by the addition of 2 µL of anti-CD3 and 5 µL of anti-CD28 mAb beads per well for 3 days. Subsequently, T-cell proliferation was measured by flow cytometry. The concentrations of IFN-γ secreted into the supernatants were determined by ELISA (MabTech, Stockholm, Sweden).

### Flow cytometry

The Abs used for flow cytometry were FITC-, PE-, or APC-conjugated mouse anti-human CD3, IFNγ, CD66b, CD16, PDL1, and CD62L Abs from BioLegend. The cultured cells were collected, washed twice, and resuspended in 100 µL of PBS containing 0.1% BSA. These cells were stained and labeled with either specific Abs or the appropriate isotype controls. The cells were incubated on ice for 30 min, washed with PBS containing 0.1% NaN_3_ and 0.5% BSA, and then fixed in 1% paraformaldehyde solution. Analyses were performed using FACScan and CellQuest software (BD Biosciences).

### Western blot

Equal amounts of protein (30–60 μg) from each sample were separated by 10% SDS-PAGE and transferred to PVDF membranes (Millipore, Boston, MA). Then, the proteins were incubated overnight at 4 °C with primary antibodies against human caspase 3 (Cell Signaling Technology, Beverly, MA) or GAPDH (Cell Signaling Technology, Beverly, MA). The membranes were washed for 30 min and then incubated with HRP-conjugated secondary antibodies for 1 h at room temperature. After the membranes were washed again for 30 min, they were visualized by enhanced chemiluminescence (ECL; Millipore, Billerica, MA) and recorded on Kodak film.

### Immunohistochemistry

The tissues were fixed in 4% paraformaldehyde (PFA; Sigma, St. Louis, MO), embedded in paraffin, and cut into 4-µm-thick sections. Endogenous peroxidase activity was blocked, antigen retrieval was performed, and the slides were stained with primary antibodies against CD66b (Abcam, Cambridge, MA) and α-SMA, which were used at the dilutions indicated by the manufacturer. Immunoreactions were detected by the Dako-Cytomation Envision HRP System (Dako, Glostrup, Denmark), and the sections were counterstained with hematoxylin (Sigma, St. Louis, MO). Negative controls were only stained with the secondary antibody. CAFs were identified by α-SMA immunoreactivity and neutrophils were identified by CD66b immunoreactivity.

### Quantitative reverse-transcription PCR

Total RNA was extracted from neutrophils using the Trizol reagent (Invitrogen). The cDNAs were synthesized by using a reverse transcription kit according to the manufacturer’s instruction (Vazyme, Shanghai, China). Quantitative reverse-transcription PCR was performed using human-specific primers for the quantification of IL-8, CCL2, and TNFa (Table 1). β-actin was used as an internal control. Reactions were performed using SYBR-Green PCR mix (Applied Biosystems, Shanghai, China) in the Bio-Rad CFX96 Real-Time System.

**Table 1:** Primer sequences of target genes

Genes	Primer sequence (50–30)	Amplicon size (bp)	Annealing temp (°C)
Mcl-1	For: ACGGCCTTCCAAGGCAT	103	64
	Rev: TTGTTACGCCGTCGCTGA		
Fas	For: TCGGAGGATTGCTCAACAAC	83	63
	Rev: ATGATGCAGGCCTTCCAAGT		
IL8	For: GCTCTGTGTGAAGGTGCAGTTT	144	61
	Rev: TTCTGTGTTGGCGCAGTGT		
CCL2	For: GAACCGAGAGGCTGAGACTA	151	53
	Rev: GCCTCTGCACTGAGATCTTC		
TNFa	For: CCGAGTGACAAGCCTGTAGC	493	56
	Rev: AGGAGGTTGACCTTGGTCTG		
β-actin	For:CACGAAACTACCTTCAACTCC	265	54
	Rev: CATACTCCTGCTTGCTGATC		

### Statistical analysis

The results are expressed as the means ± SEM. The statistical significance of differences between groups was determined by Student’s *t*-tests. SPSS statistical software (version 13.0) was used for all statistical analyses. All data were analyzed using two-tailed tests unless otherwise specified, and *P* < 0.05 was considered statistically significant. *P ≤ 0.05, **P ≤ 0.01, ***P ≤ 0.001.
